# Retraction: IL-6 Promotes Cancer Stemness and Oncogenicity in U2OS and MG-63 Osteosarcoma Cells by Upregulating the OPN-STAT3 Pathway

**DOI:** 10.7150/jca.67290

**Published:** 2021-10-08

**Authors:** Journal of Cancer

This article (IL-6 Promotes Cancer Stemness and Oncogenicity in U2OS and MG-63 Osteosarcoma Cells by Upregulating the OPN-STAT3 Pathway. Chuan Zhang, Kun Ma, Wu-Yin Li. *J Cancer* 2019; 10(26):6511-6525) has been retracted due to concerns on multiple image duplications as identified by PubPeer users. The corresponding author has been contacted by the journal, and the authors expressed apology for their negligence and mistakes in this paper.

